# Evaluation and Comparison of the Efficacy of Subcutaneous and Sublingual Immunotherapy for the Treatment of Allergic Asthma in Children

**DOI:** 10.3390/children11060692

**Published:** 2024-06-05

**Authors:** Vojko Berce, Maša Cugmas, Staša Čopi, Brigita Koren, Maja Tomazin, Tina Hojnik

**Affiliations:** 1 Department of Pediatrics, University Medical Centre Maribor, Ljubljanska Ulica 5, 2000 Maribor, Slovenia; brigita.koren@ukc-mb.si (B.K.); maja.tomazin@ukc-mb.si (M.T.); tina.hojnik@ukc-mb.si (T.H.); 2 Pediatrija Šentilj Outpatient Clinic, Pod Hribom 14, 2212 Municipality of Sentilj, Slovenia; masa.cugmas@student.um.si; 3 Department of Pediatrics, General Hospital Ptuj, Potrceva Cesta 23, 2250 Ptuj, Slovenia; stasa.copi@sb-ptuj.si

**Keywords:** sublingual immunotherapy, subcutaneous immunotherapy, efficacy, allergic asthma, children

## Abstract

Specific immunotherapy represents the only potentially curative treatment for allergic asthma. Allergens can be administered subcutaneously (SCIT) or sublingually (SLIT). The aim of the current study was to evaluate and compare the efficacy of SCIT and SLIT for the treatment of allergic asthma in children. Our study included 69 children with allergic asthma who underwent immunotherapy for house dust mites or pollen for at least 3 consecutive years. After 3 years of SCIT and SLIT, the median number of asthma exacerbations in the last three months decreased from 2 to 0 (*p* < 0.01) and from 1 to 0 (*p* < 0.01), respectively. When comparing the efficacy of SCIT and SLIT, our study revealed a significantly better efficacy of SCIT only in terms of increasing lung function. The median increase in forced expiratory volume in one second (FEV1) after 3 years was 8% with SCIT and −1% with SLIT (*p* < 0.01). Daily controller therapy could be withdrawn or reduced in 9 out of 16 (56.3%) children who received it before SCIT (*p* < 0.01) and in 19 of 29 (65.6%) children who received it before SLIT (*p* < 0.01), but the difference in efficacy was not significant (*p* = 0.88). Both SCIT and SLIT are effective treatments for allergic asthma in children.

## 1. Introduction

Specific immunotherapy (SIT) currently represents the only potentially curative treatment for allergic respiratory diseases, especially allergic rhinitis and allergic asthma [1]. Patients can receive treatment subcutaneously (SCIT) or sublingually (SLIT) in the form of either tablets or solutions (drops) of the airborne allergen at a specific concentration. In both cases, the allergen is introduced into the body, first to establish tolerance to the inhaled allergen and then to stimulate regulatory B and T cells, which results in the suppression of allergen-specific T cells and a switch in B cells from the secretion of allergen-specific class E antibodies (sIgE) to the production of inert immunoglobulin class G4 (IgG4) antibodies, which bind to the allergen but do not cause mast cell degranulation. Antigen-presenting cells (APCs) play an important role in immunotherapy, so immunotherapeutic agents should be applied where they are abundant (oral mucosa, subcutaneous tissue). The assumed mechanism is particularly effective if immunotherapy is given at regular intervals and at the same dose (allergen concentration) for several years [1,2].

After several months of SIT, the immune responses in the airway mucosa after airborne allergen exposure are diminished [3,4]. SIT for house dust mites (HDM) and pollen reduce airway hyperresponsiveness to allergen exposure, improve asthma control, reduce the need for anti-asthmatic therapy and prevent further sensitization [5,6]. Compared to SLIT, SCIT has been shown to have a more pronounced effect on IgG4-specific antibody production and a slightly faster clinical impact [2]. However, SLIT for (HDM) has also been found to have a beneficial effect on biomarkers of allergic inflammation [7]. The early introduction of SLIT in children with allergic upper airway disease effectively prevented the development of asthma. For the effect of SIT to last for at least several years after cessation, both SLIT and SCIT immunotherapy should be given for at least 3 years [8].

The results of studies comparing the efficiency of SCIT and SLIT in the treatment of allergic asthma in children are somewhat conflicting, but at least for HDM immunotherapy, SCIT has a slightly faster and better effect on asthma symptoms than SLIT [9]. The European Academy of Allergy and Clinical Immunology (EAACI) suggested the number of asthma exacerbations or the time from the discontinuation of anti-asthmatic therapy to the first asthma exacerbation as a criterion for the effectiveness of SLIT [10].

The Global Initiative for Asthma (GINA) recommends HDM immunotherapy for adults and adolescents (>12 years) with allergic asthma and concomitant allergic rhinitis and whose sensitization to HDM contributes to asthma exacerbation. Because of the potential side effects of SCIT, the GINA recommendations refer to SLIT as an adjunctive therapy in patients with mild to moderate asthma (steps two, three and four) [11]. On the other hand, the US National Asthma Education and Prevention Program (NAEPP) guidelines recommend SCIT to HDM, pollen or animal epithelium in patients with mild to moderate asthma who are more than 5 years old and in whom allergen exposure triggers the exacerbation of asthma [12].

Due to the inconsistencies between the different international guidelines and the lack of studies comparing the efficacy of SCIT and SLIT for the treatment of allergic asthma in children based on the efficacy indicators recommended by the EAACI [10], the primary aim of the study was to directly compare the efficacy of SCIT and SLIT in the treatment of allergic asthma in children. The study objective was to evaluate and compare the effects of SCIT and SLIT for HDM and grass and/or tree pollen on lung function, airway inflammation, asthma control, the number of exacerbations and the need for daily controller therapy in children with allergic asthma. In addition, this study aimed to compare the significant side effects of SCIT and SLIT in children with allergic asthma.

The hypothesis of the study was that SCIT would be more effective than SLIT at improving asthma control, improving lung function, reducing the number of exacerbations and reducing or discontinuing daily controller anti-asthmatic therapy.

In addition, this study hypothesized that patients with SCIT would have more side effects, making it necessary to discontinue SCIT.

## 2. Materials and Methods

### 2.1. Participants

The present study included all patients with mild to moderate allergic asthma who were treated at our allergy outpatient clinic from 1 January 2019 to 31 December 2023 and who received immunotherapy for HDM or trees and/or grass pollen for at least three consecutive years.

The diagnosis of asthma was made in line with the European Respiratory Society (ERS) criteria [13]. Immunotherapy was not administered to patients with poor lung function, defined as a lung volume in the first second of forced exhalation (FEV1) less than 70% of the predicted value.

The children were aged 5–16 years at the start of immunotherapy. Only patients sensitized to HDM or pollens (see Section 2.2. below) whose asthma symptoms were triggered by allergen exposure and who exhibited eosinophilic inflammation (proven by fractional exhaled nitric oxide [FeNO] levels above 25 parts per billion (ppb) on at least one occasion before the initiation of immunotherapy [13]) were considered to have allergic asthma and were enrolled in the study. The number of asthma exacerbations before the introduction of immunotherapy and after 3 years of immunotherapy was documented. Thus, for HDM immunotherapy, the number of asthma exacerbations in the last three months before the introduction of immunotherapy was recorded, and for pollen immunotherapy, the number of exacerbations in the last flowering season before the introduction of immunotherapy and then again in the last three months of the third year of immunotherapy (for HDM) and in the third flowering season of immunotherapy (for pollen) were recorded. An exacerbation was considered to have occurred if patients reported episodes of worsening asthma symptoms and a fall in peak expiratory flow (PEF) of at least 20% from the baseline, requiring the regular use of asthma relievers for at least one day [11,12].

Asthma control was also evaluated using the Asthma Control Test (ACT) questionnaire, and scores were recorded before and after 3 years of immunotherapy. For patients receiving pollen immunotherapy, the assessment was carried out during the flowering season [14]. Furthermore, daily controller anti-asthmatic therapy before and after 3 years of immunotherapy was documented.

This study excluded children who stopped immunotherapy prematurely or who had received it for less than 3 years.

### 2.2. Methods

All children underwent skin prick testing for airborne allergens using Lofarma (Milan, Italy) tests and/or the measurement of specific immunoglobulin class E (sIgE) levels for airborne allergens with the ImmunoCAP system (Phadia, Uppsala, Sweden). The presence of a wheal at least 3 mm larger in diameter compared with the negative control and/or an sIgE level > 0.35 IU/mL were considered evidence of sensitization.

Spirometry and FeNO measurements were also performed in all children using a Ganshorn SpiroScout spirometer (Ganshorn Medizin Electronic GmbH, Niderlauer, Germany). Spirometry was performed before the start of immunotherapy and then annually thereafter. The FEV1 (as a percentage of the reference value [15]), the ratio of the FEV1 to the forced vital capacity (FVC) or the Tiffeneau index (TI) and the FeNO value were recorded both before the initiation of immunotherapy and after 3 years of immunotherapy. In patients with pollen allergies, measurements were performed during the flowering season. All the measurements were performed according to the European Respiratory Society (ERS) guidelines [13,16].

Sublingual immunotherapy for HDM was performed on a daily basis throughout the year with Acarizax 12 SQ tablets (ALK Abello, Horsholm, Denmark) for children over 12 years of age. For children under 12 years, Sublivac solution (HAL Allergy Group, Leiden, The Netherlands) or Staloral solution (Stallergens, Antony, Hauts de Seine, France) was used. Sublingual immunotherapy to grass pollen was performed with Oralair tablets (Stallergens, Antony, Hauts de Seine, France), and immunotherapy to tree pollen was performed with Sublivac solution (HAL Allergy Group, Leiden, The Netherlands) or Staloral solution (Stallergens, Antony, Hauts de Seine, France). Sublingual immunotherapy to pollen was performed daily but seasonally, at least four months before and during the flowering season.

Subcutaneous immunotherapy for HDM was performed year-round with Purethal (HAL Allergy Group, Leiden, The Netherlands) and with tree and/or grass pollen seasonally, at least 4 months before and during the flowering season, as well as with Purethal (HAL Allergy Group, Leiden, The Netherlands). After the initial build-up phase, allergy injections were given every 4–6 weeks.

### 2.3. Ethical Approval and Data Availability

The study received approval from the Ethics Committee of the University Clinical Center Maribor (approval number: UKC-MB-KME-1/24, issued 15 January 2024) and was conducted in accordance with the Declaration of Helsinki (1975), revised in Edinburgh in 2000. Since all participants were under 16 years old at the start of immunotherapy, their legal guardians provided informed consent. The raw data utilized in this study are freely accessible at Kaagle at https://www.kaggle.com/datasets/vojkoberce/scit-slit-raw-data-ii (accessed on 4 March 2024).

### 2.4. Statistical Analysis

Statistical analysis was conducted using IBM SPSS 26.0 software (IBM Inc., Chicago, IL, USA). The Kolmogorov–Smirnov test was first used to check the normality of the distribution. Initially, data from patients who underwent SCIT or SLIT before beginning immunotherapy were compared. The Mann—Whitney U test was used to analyze quantitative variables, whereas the chi—square test or Fisher’s exact test was used to analyze qualitative variables.

The efficacy of immunotherapy within each group (paired samples) was assessed by comparing quantitative variables before and after immunotherapy in the same patient using the Wilcoxon signed-rank test. For the comparison of the efficacy of SCIT and SLIT, the percentage of the increase/decrease in quantitative variables (such as the FEV1, TI, FeNO and ACT questionnaire score) after immunotherapy was calculated, and these values were compared using the Mann—Whitney U test. This test was also used for analyzing the difference between the number of asthma exacerbations before and after immunotherapy. Regarding daily controller therapy, patients were categorized post-immunotherapy into groups with lower, equal or higher doses compared to pre-immunotherapy doses, and this difference was considered an ordinal variable and compared between both groups (SCIT and SLIT), again using the Mann—Whitney U test. A *p* value below 0.05 was considered statistically significant.

## 3. Results

### 3.1. Epidemiological and Clinical Characteristics

The current study enrolled 69 children, of whom 24 (34.8%) received SCIT, 45 (65.2%) received SLIT and 23 (33.3%) were female. The median age at immunotherapy initiation was 11 years, and the median duration of immunotherapy was 3 years for both the SCIT and SLIT patients. House dust mite immunotherapy was performed in 28 (40.6%) children, and pollen immunotherapy was performed in 41 (59.4%) children. Among the 45 children with SLIT, 14 (31.1%) received immunotherapy for mites and 31 (68.9%) received it for pollen, while among the children with SCIT, 14 (58.3%) received immunotherapy for mites and 10 (41.7%) received it for pollen. All subjects had concurrent allergic rhinitis and/or conjunctivitis.

The epidemiological and clinical characteristics of the children before immunotherapy are presented in Table 1.

Before immunotherapy, 16 (66.6%) children who received SCIT and 29 (64.4%) children who received SLIT also received daily controller anti-asthmatic therapy (*p* = 0.86).

### 3.2. Efficacy of Sublingual and Subcutaneous Immunotherapy

The efficacy of immunotherapy was evaluated separately for SCIT and SLIT, as presented in Table 2 and Table 3.

Regarding daily controller therapy, 9 children out of 16 (56.3%) who used daily controller therapy before SCIT were able to reduce or discontinue the use, and daily controller therapy was newly introduced during the SCIT for only one child (*p* < 0.01). In the group receiving SLIT, 19 out of 29 (65.6%) children who used daily controller therapy before SLIT were able to reduce or discontinue its use. In 5 children, an increase in the dose or newly introduced daily controller therapy was needed during SLIT (*p* < 0.01). However, the differences in the effects of SCIT and SLIT on the need for daily controller therapy were not found to be significant (*p* < 0.88). Significant adverse effects of immunotherapy (requiring the discontinuation of SIT) were reported by 2 (8.3%) children with SCIT and 7 (15.5%) children with SLIT (*p* = 0.33). Of these, one patient with SLIT experienced anaphylaxis (at the first administration), and in one patient, SLIT probably caused eosinophilic esophagitis.

### 3.3. Comparison of the Efficacy of Subcutaneous and Sublingual Immunotherapy

Our study also compared the efficacy of SLIT and SCIT in terms of other clinical parameters, as shown in Table 4.

**Figure 1:**
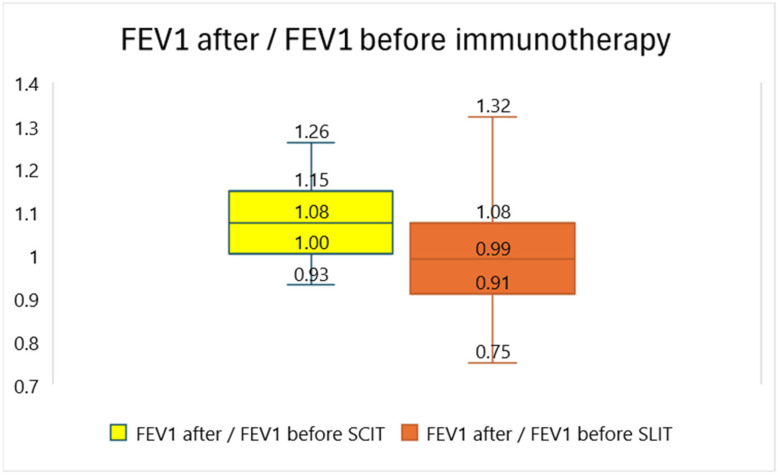
Comparison of the efficacy of subcutaneous and sublingual immunotherapy regarding lung function. FEV1—forced expiratory volume; SCIT—subcutaneous immunotherapy; SLIT—sublingual immunotherapy. The ratios of the percentage of predicted FEV1 before and after 3 years of immunotherapy are presented. The numbers in the boxplot represent (from above) the maximum, third quartile, median, first quartile and minimum of the distribution of the ratios of FEV1 before and after immunotherapy.

A comparison of the effects of SCIT and SLIT on lung function is presented in Figure 1.

Furthermore, the efficacies of SCIT and SLIT for house dust mite immunotherapy and pollen immunotherapy were compared, as shown in Table 5 and Table 6.

The discontinuation of or reduction in daily controller therapy was achieved in four (28.6%) patients who underwent SCIT and in five (33.3%) patients who underwent SLIT for house dust mites (*p* = 0.50).

Daily controller therapy (during the flowering season) could be stopped or reduced in 5 (50%) children receiving SCIT and in 14 (45.2%) children receiving SLIT for pollen. However, an increase in the dose of daily controller therapy was needed for one child (10%) treated with SCIT and five (14.3%) treated with SLIT. The difference between the SCIT and SLIT groups for pollen in terms of the need for daily controller therapy was not significant (*p* = 0.89).

## 4. Discussion

The originality and added value of this study are mainly in the assessment and comparison of the efficacy of SCIT and SLIT for the treatment of allergic asthma in children, which are based on the EAACI recommendations for the assessment of the efficacy of SIT in the treatment of asthma [10]. This study revealed that both subcutaneous and sublingual immunotherapy may be effective adjunctive treatments for patients with allergic asthma. The potential beneficial effect of both immunotherapy modalities is reflected by a reduction in the exacerbation rate, the better control of asthma (according to the ACT questionnaire) and a reduction in the need for daily controller therapy. However, the effect of immunotherapy on reducing eosinophilic inflammation in the airways (as measured by the FeNO value) was not statistically significant. Regarding lung function (FEV1), there was a significant improvement only with SCIT but not with SLIT.

The results of the present study are mostly in line with the findings of similar comparative studies. Thus, Chelladurai et al. performed a meta-analysis and found that both immunotherapy modalities were effective in adults with allergic asthma, highlighting the superior effect of SCIT (compared with SLIT) on asthma symptoms [17]. A meta-analysis conducted by Ma et al. also demonstrated an effect of SLIT on asthma symptoms and no effect on FeNO or FEV1 in adults with allergic asthma. In contrast to the present study, they found no impact of SLIT on the need for daily controller therapy with inhaled corticosteroids. However, unlike the present study, the meta-analysis performed by Ma et al. did not include patients with SCIT and therefore did not compare the two treatment modalities [18]. In a review article derived from several comparative meta-analyses, Tsabouri et al. also reported a slightly faster and more pronounced effect of SCIT (compared with SLIT) in the treatment of allergic asthma in children [9]. However, a meta-analysis performed by Lin et al. confirmed the significant effect of SLIT on the need for daily controller therapy, which is in line with the results of this study, but in addition (and unlike the results of this study), they reported a positive effect of SLIT on FEV1 [19]. However, the meta-analysis performed by Dhami et al. revealed no effect of SCIT or SLIT on asthma control, lung function or exacerbation rates in adults with asthma [20]. The greater efficacy of immunotherapy found in the present study may indicate a better response to immunotherapy for airborne allergens in children compared to the adult population. The marginally better effect of SCIT (compared to SLIT) on the number of asthma exacerbations in this study may also be attributed to the higher number of exacerbations in the SCIT group before the introduction of immunotherapy. Regarding the effect of SIT on lung function (FEV1), it should be emphasized that the median FEV1 values were within normal limits (99% of predicted in the SCIT group and 97% of predicted in the SLIT group) even before immunotherapy, as only patients with mild or moderate asthma were included. Therefore, the observed increase in FEV1 (median 8%) after SCIT, compared to no increase with SLIT, is not clinically relevant for most patients.

This study also compared the efficacy of SLIT and SCIT separately for HDM and pollen and revealed more differences in favor of SCIT for HDM immunotherapy than for pollen immunotherapy. Thus, a significantly greater decrease in FeNO and increase in the TI were found in patients receiving SCIT than in those receiving SLIT for HDM. The effect of SCIT for HDM on the reduction of asthma exacerbations in the present study was also more pronounced than that of SLIT, although the median reduction in asthma exacerbations with both treatments was 1. The differences in efficacy between SCIT and SLIT were smaller for pollen immunotherapy, where only a significantly greater improvement in lung function (FEV1) was observed with SCIT than with SLIT, but we found no difference between SCIT and SLIT in terms of symptom improvement or reduction in exacerbations. In addition, there was no difference in efficacy between both treatment modalities regarding the discontinuation of daily controller therapy, which applies to both pollen and HDM immunotherapy. Most similar comparative studies have thus far been performed in patients with allergic rhinitis, where objective measurements are more difficult to perform than in patients with asthma. Thus, Brüggenjürgen et al. reported the greater efficacy (in terms of symptom reduction) and cost-effectiveness of SCIT (compared to SLIT) for grass pollen for the treatment of hay fever [21]. In contrast, Yang et al. reported at least an equivalent efficacy of SLIT compared with that of SCIT in children with allergic upper airway disease and SIT for either grass pollen or HDM [22].

Karakoc-Aydiner et al. performed a randomized controlled trial in children with mild asthma who were allergic to HDM. This study demonstrated that SCIT and SLIT for HDM were similarly and significantly effective at reducing medication usage, which was confirmed in the present study [23]. A review article by Richards et al. reported a significant effect of SLIT for HDM on the reduction of asthma symptoms, which is comparable to that of SCIT, and a slightly weaker effect of SLIT (compared to that of SCIT) in terms of reducing the need for daily controller therapy [24]. These results are somewhat at odds with the results of the present study, which revealed that SCIT and SLIT for HDM had comparable effects on reducing the need for daily controller therapy and that SLIT had a significantly inferior effect on reducing asthma exacerbations. Similar findings were reported by Kim et al. and Hamada et al. in adults with perennial allergic rhinitis and allergy to HDM. Both studies confirmed the efficacy of SLIT and SCIT for HDM but revealed that SCIT was slightly superior to SLIT [25,26]. The better efficacy of SCIT (compared to SLIT) for HDM in this study can be partially attributed to the high proportion of children in whom SLIT for HDM was performed with drops and SLIT for grass pollen was performed with tablets. Some data from the literature suggest that the efficacy of tablet-based SLIT is greater than that of droplet-based SLIT [27]. Regarding the effect of SLIT for HDM on FeNO, the results of this study are in line with the findings of Parisi et al., who reported a significant reduction in nasal NO and nasal eosinophils but no significant effect of SLIT for HDM on FeNO. They also concluded that nasal NO and nasal eosinophils are important predictors of SLIT efficacy, which we did not analyze in this study [28]. The more profound effect of SCIT (compared to SLIT) for HDM on FeNO could be attributed to the much broader effect of SCIT on the molecular level. According to the mechanism presented by Cuppari et al., both SLIT and SCIT reduce eosinophilic inflammation in the nasal mucosa and induce the production of blocking IgG4 antibodies. However, unlike SCIT, SLIT does not affect dendritic cells and T lymphocytes in the oral mucosa and therefore has much lower anti-inflammatory potential outside the upper airways [29].

The proportion of children experiencing significant side effects who required the discontinuation of SIT was comparably low in both groups. These observations align with previous reports, although Bauer et al. reported a slightly higher number of systemic reactions with SCIT compared to those with SLIT [30]. Unfortunately, we did not record and compare compliance with SIT. However, the total dropout rate was low in both treatment groups.

The present study has several limitations and weaknesses, including the relatively small number of subjects and heterogeneity (seasonal or persistent asthma, SLIT tablets or drops, immunotherapy to different pollens). In particular, the number of subjects in each subgroup is too small (e.g., SCIT for pollen was performed in only 10 subjects), so for individual allergens (e.g., dust mites or pollen), the results on the comparative efficacy of SLIT and SCIT should be taken with reservation, and randomized controlled trials on larger samples are needed to confirm these results. The generalizability of the results of the present study may be limited by potential selection bias, which is characteristic for observational studies. An additional limitation of the present study is the incomplete equivalence of patients in the two groups (SCIT, SLIT) regarding the number of asthma exacerbations in the 3 months (or last season) before the initiation of immunotherapy, such that the median number of exacerbations before the initiation of SCIT was two and that before the initiation of SLIT was one. The present study mainly included patients with mild asthma and normal lung function; therefore, conclusions on the effect of SIT on lung function based on our results are limited. In addition, the present study did not test and compare the sustainability of the effect of SIT; thus, no conclusions regarding this issue can be made. We did not analyze the effect of SIT on the prevention of further sensitizations and comorbidities, although such effects have been reported previously [31]. Due to the heterogeneity of anti-asthmatic therapies (different inhaled corticosteroids, often in fixed combination with long-acting beta2 agonists), the exact equivalent dose of daily controller therapy could not be accurately standardized.

Further studies are likely to evaluate and compare alternative routes, such as intradermal and intralymphatic [29] routes, as well as allergen presentation platforms, including novel adjuvants or delivery systems, and SIT performed with selected allergen components.

## 5. Conclusions

In summary, both sublingual and subcutaneous immunotherapy can be effective therapeutic tools for children with allergic asthma, mainly because of their beneficial impacts on asthma symptoms, on exacerbations and on the need for daily controller therapy. Considering the relative ease and painless administration of SLIT in the home environment and the comparable incidence of severe side effects with SCIT, we can conclude that SLIT represents a suitable alternative for the adjunctive treatment of allergic asthma in children, which is applicable to house dust mite allergy but even more so to seasonal asthma and pollen allergy.

## Figures and Tables

**Table 1:** Epidemiological and clinical characteristics of children with asthma before immunotherapy.

Characteristic [N (%)] ^1^	SCIT (*N* = 24)	SLIT (*N* = 45)	*p* Value
Female sex	6 (25.0)	17 (34.7)	0.21
Comorbidities (atopic dermatitis, food allergy)	6 (25.0)	15 (30.6)	0.33
Daily controller therapy	16 (66.6)	29 (59.2)	0.53
**Characteristic [median (IQR)]**			
Age (years)	11.5 (9.5–14.0)	11 (8–13)	0.17
FeNO (ppb)	43 (20–84)	37 (18–66)	0.39
FEV1 (% of predicted)	99 (89.5–102.5)	97 (88.5–104.5)	0.98
TI (%)	88 (83–91)	89 (82–92)	0.41
ACT score	23 (22–24)	24 (22–25)	0.09
Number of exacerbations ^2^	2 (1–3)	1 (0–2)	0.02
Duration of immunotherapy (years)	3 (3–4)	3 (3–4)	0.30

^1^ Number of subjects (percentage in parentheses). ^2^ Number of asthma exacerbations in the last three months prior to the introduction of immunotherapy. SCIT—subcutaneous immunotherapy; SLIT—sublingual immunotherapy; FeNO—fractional exhaled nitric oxide—parts per billion (ppb); FEV1—forced expiratory volume (percentage of predicted reference value) [15]; TI—Tiffeneau index (ratio between FEV1 and forced vital capacity); ACT—Asthma Control Test [14]; IQR—interquartile range.

**Table 2:** Efficacy of subcutaneous immunotherapy—comparison of clinical characteristics of children with asthma before and after 3 years of immunotherapy.

Characteristic [Median (IQR)]	Before SCIT	After SCIT	*p* Value
FeNO (ppb)	43 (20–84)	23.5 (10–48)	0.09
FEV1 (% of predicted)	99 (89.5–102.5)	105.5 (98–114)	<0.01
TI (%)	88 (83–91)	86 (82–96)	0.54
ACT score	23 (22–24)	25 (24–25)	<0.01
Number of exacerbations ^1^	2 (1–3)	0 (0–1)	<0.01

^1^ Number of asthma exacerbations in the 3 months prior to the introduction of immunotherapy and after three years of immunotherapy. SCIT—subcutaneous immunotherapy; FeNO—fractional exhaled nitric oxide—parts per billion (ppb); FEV1—forced expiratory volume (percentage of predicted reference value) [15]; TI—Tiffeneau index (ratio between FEV1 and forced vital capacity); ACT—Asthma Control Test [14]; IQR—interquartile range.

**Table 3:** Efficacy of sublingual immunotherapy—comparison of clinical characteristics of children with asthma before and after 3 years of immunotherapy.

Characteristic [Median (IQR)]	Before SLIT	After SLIT	*p* Value
FeNO (ppb)	37 (18–66)	35 (18–63)	0.68
FEV1 (% of predicted)	97 (88.5–104.5)	96 (87–103)	0.38
TI (%)	89 (82–92)	87 (80–91)	0.35
ACT score	24 (22–25)	25 (24–25)	<0.01
Number of exacerbations ^1^	1 (0–2)	0 (0)	<0.01

^1^ Number of asthma exacerbations in the 3 months prior to the introduction of immunotherapy and after three years of immunotherapy. SLIT—sublingual immunotherapy; FeNO—fractional exhaled nitric oxide—parts per billion (ppb); FEV1—forced expiratory volume (percentage of predicted reference value) [15]; TI—Tiffeneau index (ratio between FEV1 and forced vital capacity); ACT—Asthma Control Test [14]; IQR—interquartile range.

**Table 4:** Comparison of the efficacy of subcutaneous and sublingual immunotherapy in children with asthma.

Characteristic [Median (IQR)]	SCIT (*N* = 24)	SLIT (*N* = 45)	*p* Value
Change in FeNO (%) ^1^	−33 (−71–+55)	−5 (−43–+92)	0.12
Change in FEV1 (% of predicted) ^1^	+8 (0–+15)	−1 (−9–+8)	<0.01
Change in TI (%) ^1^	0 (−3–+6)	0 (−6–+4)	0.36
Change in ACT score (%) ^1^	+9 (+1–+10)	+4 (0–+9)	0.13
Decrease in the number of asthma exacerbations ^2^	2 (1–3)	1 (0–2)	0.06

^1^ Percentage of increase/decrease after three years of immunotherapy. ^2^ Difference between the number of asthma exacerbations in the last three months prior to the introduction of immunotherapy and after three years of immunotherapy. SCIT—subcutaneous immunotherapy; SLIT—sublingual immunotherapy; FeNO—fractional exhaled nitric oxide—parts per billion (ppb); FEV1—forced expiratory volume (percentage of predicted reference value) [15]; TI—Tiffeneau index (ratio between FEV1 and forced vital capacity); ACT—Asthma Control Test [14]; IQR—interquartile range.

**Table 5:** Comparison of the efficacy of subcutaneous and sublingual immunotherapy for house dust mites in children with asthma.

Characteristic [Median (IQR)]	SCIT (*N* = 14)	SLIT (*N* = 14)	*p* Value
Change in FeNO (%) ^1^	−66 (−75–−12)	+27 (+26–+143)	<0.01
Change in FEV1 (% of predicted) ^1^	+8 (0–+15)	−3 (−9–+4)	0.07
Change in TI (%) ^1^	+1 (−4–+6)	−4 (−8–0)	0.02
Change in ACT score (%) ^1^	+6 (+3–+9)	+2 (0–+6)	0.16
Decrease in number of asthma exacerbations ^2^	1 (1–2)	1 (0–1)	0.02

^1^ Percentage of increase/decrease after three years of immunotherapy. ^2^ Difference between the number of asthma exacerbations in the last three months prior to the introduction of immunotherapy and after three years of immunotherapy. SCIT—subcutaneous immunotherapy; SLIT—sublingual immunotherapy; FeNO—fractional exhaled nitric oxide—parts per billion (ppb); FEV1—forced expiratory volume (percentage of predicted reference value) [15]; TI—Tiffeneau index (ratio between FEV1 and forced vital capacity); ACT—Asthma Control Test [14]; IQR—interquartile range.

**Table 6:** Comparison of the efficacy of subcutaneous and sublingual immunotherapy for pollen in children with asthma.

Characteristic [Median (IQR)]	SCIT (*N* = 10)	SLIT (*N* = 31)	*p* Value
Change in FeNO (%) ^1^	+27 (−66–+118)	−16 (−48–+65)	0.48
Change in FEV1 (% of predicted) ^1^	+10 (+1–+20)	−1 (−9–+8)	<0.01
Change in TI (%) ^1^	−1 (−3–+6)	+1 (−3–+5)	0.73
Change in ACT score (%) ^1^	+8 (0–+10)	+4 (0–+9)	0.43
Decrease in number of asthma exacerbations ^2^	1 (1–2)	1 (0–2)	0.58

^1^ Percentage of increase/decrease after three years of immunotherapy. ^2^ Difference between the number of asthma exacerbations in the last flowering season prior to the introduction of immunotherapy and in the third season after the introduction of immunotherapy. SCIT—subcutaneous immunotherapy; SLIT—sublingual immunotherapy; FeNO—fractional exhaled nitric oxide—parts per billion (ppb); FEV1—forced expiratory volume (percentage of predicted reference value) [15]; TI—Tiffeneau index (ratio between FEV1 and forced vital capacity); ACT—Asthma Control Test [14]; IQR—interquartile range.

## Data Availability

The raw data used in this study are openly available in Kaagle at https://www.kaggle.com/datasets/vojkoberce/scit-slit-raw-data-ii (accessed on 4 March 2024).
